# Glycolysis-Related Gene Analyses Indicate That DEPDC1 Promotes the Malignant Progression of Oral Squamous Cell Carcinoma via the WNT/β-Catenin Signaling Pathway

**DOI:** 10.3390/ijms24031992

**Published:** 2023-01-19

**Authors:** Guangzhao Huang, Su Chen, Jumpei Washio, Grace Paka Lubamba, Nobuhiro Takahashi, Chunjie Li

**Affiliations:** 1 State Key Laboratory of Oral Diseases, National Clinical Research Center for Oral Diseases, West China Hospital of Stomatology, Sichuan University, Chengdu 610044, China; 2 Department of Head and Neck Oncology, West China Hospital of Stomatology, Sichuan University, Chengdu 610044, China; 3 Division of Oral Ecology and Biochemistry, Tohoku University Graduate School of Dentistry, Sendai 980-8575, Japan

**Keywords:** glycolysis, OSCC, bioinformatics, DEPDC1

## Abstract

Increasing evidence suggests that aerobic glycolysis is related to the progression of oral squamous cell carcinoma (OSCC). Hence, we focused on glycolysis-related gene sets to screen for potential therapeutic targets for OSCC. The expression profiles of OSCC samples and normal controls were obtained from The Cancer Genome Atlas (TCGA). Then, the differentially expressed gene sets were selected from the official GSEA website following extraction of the differentially expressed core genes (DECGs). Subsequently, we tried to build a risk model on the basis of DECGs to predict the prognosis of OSCC patients via Cox regression analysis. Furthermore, crucial glycolysis-related genes were selected to explore their biological roles in OSCC. Two active glycolysis-related pathways were acquired and 66 DECGs were identified. Univariate Cox regression analysis showed that six genes, including HMMR, STC2, DDIT4, DEPDC1, SLC16A3, and AURKA, might be potential prognostic factors. Subsequently, a risk formula consisting of DEPDC1, DDIT4, and SLC16A3 was established on basis of the six molecules. Furthermore, DEPDC1 was proven to be related to advanced stage cancer and lymph node metastasis. Moreover, functional experiments suggested that DEPDC1 promoted the aerobic glycolysis, migration, and invasion of OSCC via the WNT/β-catenin pathway. The risk score according to glycolysis-related gene expression might be an independent prognostic factor in OSCC. In addition, DEPDC1 was identified as playing a carcinogenic role in OSCC progression, suggesting that DEPDC1 might be a novel biomarker and therapeutic target for OSCC.

## 1. Introduction

OSCC is one of the prevalent types of head and neck squamous cell carcinomas. Recently, OSCC has attracted increasing attention owing to its severe impact on patient quality of life. With advances in diagnosis and operative treatment, the prognosis and quality of life have significantly improved. However, for some advanced malignancies, the 5-year overall survival is just under 50% [1]. In addition, lymph node metastasis and local recurrence contribute to the poor prognosis. Studies have indicated that the overall survival of patients with early-stage OSCC could be improved to more than 85% [2]. Nevertheless, there are few early effective biomarkers and therapeutic targets for OSCC. Therefore, it is vital to explore the mechanism of OSCC progression and identify effective biomarkers to improve the overall survival for advanced-stage OSCC patients.

Cancer cells are characterized by infinite proliferation. Metabolism includes a variety of interconnected cellular chemical transformations that provide energy to sustain life [3]. To meet their abundant energy demands, cancer cells show metabolic plasticity in the reprogramming of metabolic pathways such as glycolysis [4]. In 1956, Warburg indicated that cancer cells performed glycolysis to acquire energy even under aerobic conditions, which is called the Warburg effect or aerobic glycolysis [5]. Aerobic glycolysis leads to an increase in glucose uptake and accumulation of lactate in cancer cells. Meanwhile, it also speeds up the production of adenosine triphosphate (ATP) [6]. Studies have demonstrated that a faster energy supply through aerobic glycolysis contributed to the proliferation of cancer cells, and the acidic microenvironment formed by accumulation of lactate also facilitated cancer invasion and metastasis [7]. At present, many studies have shown that aerobic glycolysis plays a crucial role in a variety of cancers. For instance, the activation of aerobic glycolysis in pancreatic cancer cells was related to their growth [8]. In addition, accelerated glycolysis also promoted the metastasis of breast cancer [9]. In OSCC, previous studies have indicated that lactate dehydrogenase A, which catalyzes the conversion of L-lactate to pyruvate, could promote malignant progression and the epithelial–mesenchymal transition (EMT) by facilitating glycolysis [10]. Meanwhile, activation of aerobic glycolysis induced by protein disulfide isomerase family 6 promoted the progression of OSCC [11]. In addition, aerobic glycolysis was also relevant to the treatment of OSCC with compounds such as Tanshinone IIA [12] and Nobiletin [13]. Therefore, aerobic glycolysis may be significantly associated with tumorigenesis, progression, and drug therapy in OSCC. However, there are few studies which focus on glycolysis-related pathways and genes to explore their prognostic values. 

In our study, we focused on glycolysis-related pathways and carried out Cox regression analysis to explore their prognostic value. As a result, the glycolysis-related biomarker DEP domain containing 1 (DEPDC1), which is a part of a transcription repressor complex and a newly discovered tumor-associated gene [14], was identified as being associated with the prognosis of OSCC. Moreover, the knockdown of DEPDC1 significantly inhibited migration, invasion, glucose uptake and accumulation of lactate. In addition, we demonstrated that DEPDC1 promoted OSCC progression via the WNT/β-catenin signaling pathway. These findings suggest that DEPDC1 may be a viable glycolysis-related biomarker and therapeutic target for OSCC.

## 2. Results

### 2.1. Glycolysis-Related Gene Sets in OSCC

**Figure 1:**
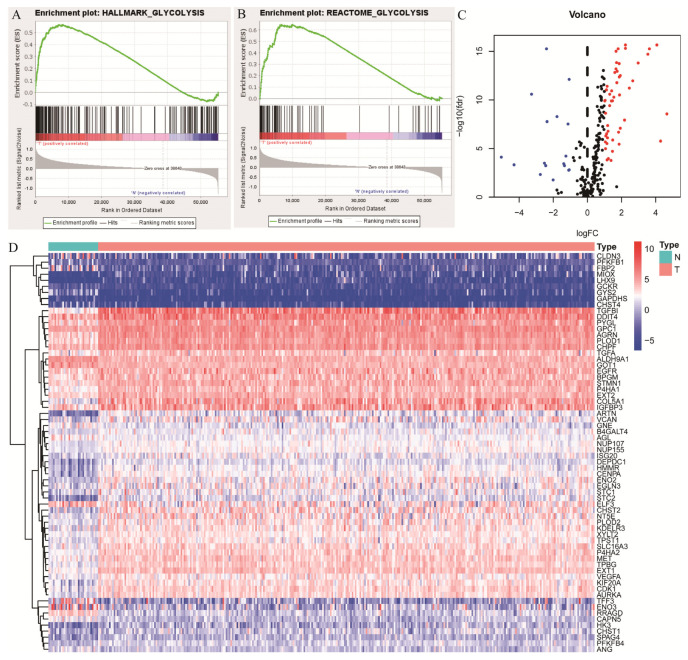
Gene set enrichment analysis (GSEA) screening of glycolysis-related gene sets. (**A**,**B**); Hallmark−glycolysis and reactome−glycolysis were significantly activated in OSCC. Higher hallmark−glycolysis and reactome−glycolysis activation were positively related to tumor samples. (**C**,**D**), The heatmap and volcano plot of differentially expressed core genes (DECGs) in these 2 gene sets. Blue and red stand for downregulation and upregulation, respectively.

The gene expression profiles and corresponding clinical data of 319 OSCC samples and 32 normal controls were obtained from TCGA. In addition, four glycolysis-related pathways including the biocarta-glycolysis-pathway, kegg-glycolysis-gluconeogenesis, hallmark-glycolysis, and reactome-glycolysis were gained from the official GSEA website. Bioinformatics analyses indicated that the gene sets hallmark-glycolysis (Figure 1A), and reactome-glycolysis (Figure 1B) were significantly activated in OSCC. To explore the prognostic value of the two gene sets, the core genes were screened out (Table 1). Furthermore, differential analyses indicated that there were 66 DEGs including 19 downregulated and 49 upregulated genes in OSCC (Figure 1C,D, Table 2 and Table 3).

### 2.2. Identification of Prognostic Cox Model

**Figure 2:**
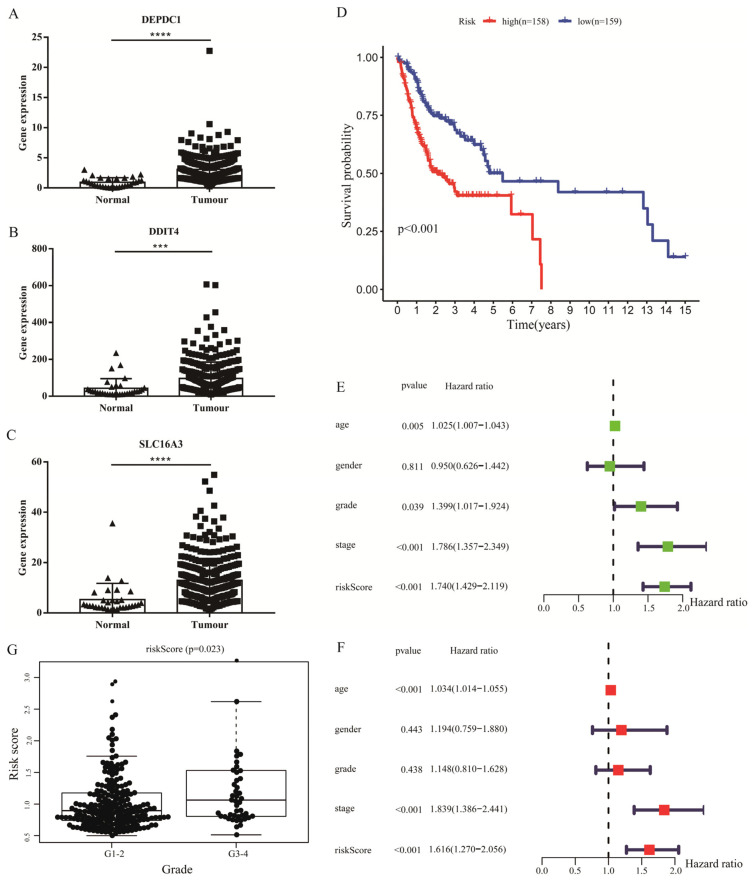
Identification of prognostic Cox model. The expression level of 3 genes, DEPDC1 ((**A**), *p* < 0.0001), DDIT4 ((**B**), *p* = 0.0008) and SLC16A3 ((**C**), *p* < 0.0001), included in the risk Cox formula in TCGA. Triangle and square stand for gene expression level in normal and tumor samples, respectively. (**D**); Survival analysis between high- and low-risk levels according to risk scores calculated from the DEPDC1, DDIT4 and SLC16A3 expression profiles of each patient. (**E**,**F**), Univariate and multivariate Cox regression analyses on the basis of risk score. (**G**), High risk score might mean poor grade level in OSCC. Significant differences were considered at *p* < 0.001 ***; and *p* < 0.0001 ****.

To investigate the prognostic value of glycolysis-related gene sets, 66 DEGs were enrolled in univariate Cox regression, which indicated that six molecules might be candidate biomarkers (Table S1). Furthermore, multivariate Cox regression and stepwise Cox analyses were used to construct the prognostic model based on the expression of six molecules. The results showed that three genes, including DNA damage inducible transcript 4 (DDIT4), DEPDC1 and solute carrier family 16 member 3 (SLC16A3), were all verified to be upregulated in TCGA OSCC samples (Figure 2A–C), and the risk-score= (DDIT4× 0.0022) + (DEPDC1× 0.0788) + (SLC16A3× 0.0316) (Table S2). Subsequently, to explore the prognostic value of the Cox model, we divided OSCC samples into high- and low-risk groups according to the median value of the risk score. Survival analysis showed that low-risk group patients had better prognosis than high-risk group patients (Figure 2D). In addition, similar results were also observed in stratification analyses (Figure S1). Moreover, the risk score might be visualized as an independent prognostic factor, in line with the univariate and multivariate Cox regression analyses (Figure 2E,F). Meanwhile, a high risk score meant poor differentiation in OSCC (Figure 2G). To intuitively understand the difference between high- and low-risk groups, the expression of risk genes combined with survival time of each patient was visualized in R software (Version R4.2.1, Chengdu, China) (Figure S2A–C). 

### 2.3. DEPDC1 Was Identified as a Potential Biomarker in OSCC

**Figure 3:**
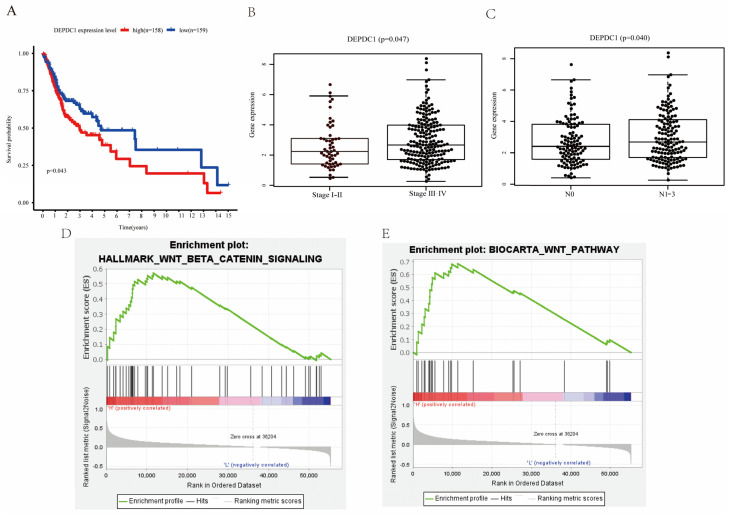
DEPDC1 was identified as a potential biomarker. (**A**), Survival analysis showed that high DEPDC1 expression levels meant poor overall survival. (**B**,**C**), DEPDC1 expression level was related to advanced stage and lymph node metastasis in OSCC. (**D**,**E**), GSEA suggested that high DEPDC1 expression was associated with higher activation of the WNT/β−catenin signaling pathway.

To further understand the prognostic value of the three risk genes, survival analyses were carried out according to the expression of each gene. The results indicated that patients with high DEPDC1 expression had worse overall survival (Figure 3A). Meanwhile, high expression of DEPDC1 was related to lymph node metastasis (Figure 3B) and advanced stage (Figure 3C). Therefore, DEPDC1 might be an advanced biomarker in OSCC. To further explore the potential mechanism of DEPDC1, we performed GSEA and showed that high expression of DEPDC1 was significantly relevant to the activation of the WNT/β-catenin signaling pathway (Figure 3D,E). In addition, the carcinogenic roles of DEPDC1 in OSCC were further explored via GSEA (Figure S3). 

### 2.4. Knockdown of DEPDC1 Inhibited the Migration and Invasion of OSCC

**Figure 4:**
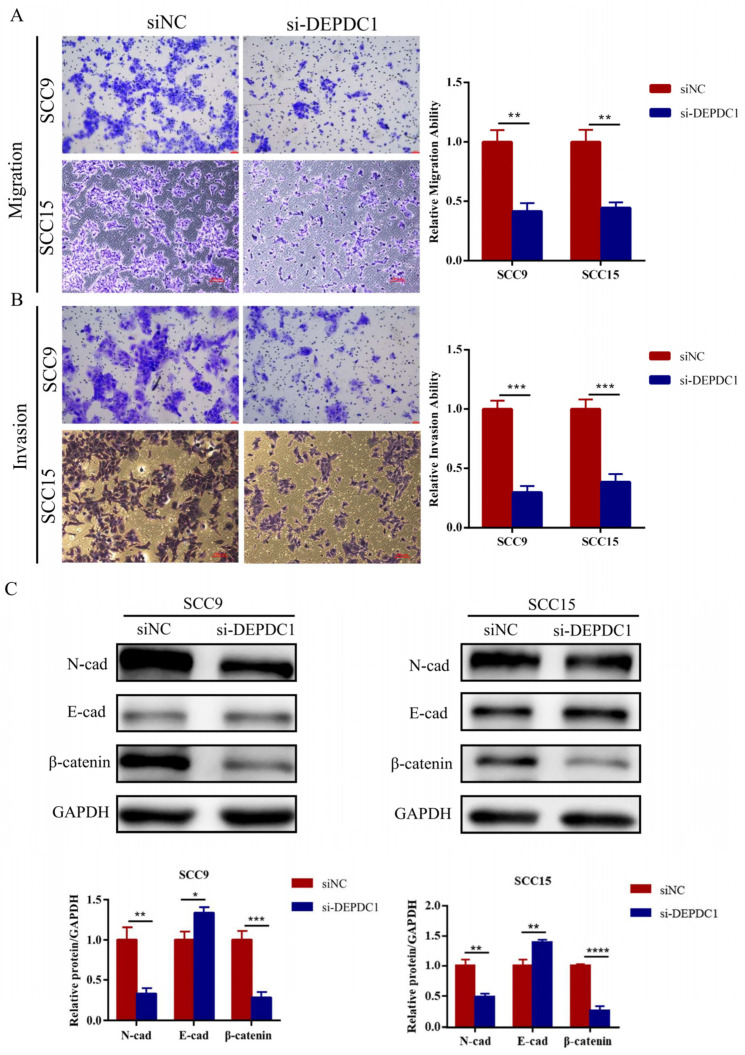
Knockdown of DEPDC1 inhibited the migration and invasion of OSCC. (**A**); (**B**), The migration (SCC9 *p* = 0.0012, SCC15 *p* = 0.0010) and invasion abilities (SCC9 *p* = 0.0002, SCC15 *p* = 0.0005) of SCC9 and SCC15 were inhibited after knockdown of DEPDC1. (**C**); Knockdown of DEPDC1 resulted in a decrease in N-cad (SCC9 *p* = 0.0024, SCC15 *p* = 0.0022) and β-catenin (SCC9 *p* = 0.0007, SCC15 *p* < 0.0001), and an increase in E-cad (SCC9 *p* = 0.0102, SCC15 *p* = 0.0040). Error bars in the graphs represent S.D. N-cad: N-cadherin, E-cad: E-cadherin, siNC: negative control of siRNA, si-DEPDC1: knockdown of DEPDC1. Significant differences were considered at *p* < 0.05 *; *p* < 0.01 **; *p* < 0.001 ***; and *p* < 0.0001 ****.

To determine the tumorigenic role of DEPDC1 in vitro, siRNAs targeting DEPDC1 and their corresponding negative controls were transfected into SCC9 and SCC15. The efficiency of knockdown was identified by qRT-PCR and Western blot (Figure S4A,B). As shown in the results, a decrease in DEPDC1 expression inhibited OSCC migration and invasion (Figure 4A,B). Meanwhile, knockdown of DEPDC1 decreased the protein expression level of β-catenin and N-cadherin and enhanced the expression of E-cadherin (Figure 4C).

### 2.5. DEPDC1 Promoted the Progression of OSCC via the WNT/β-Catenin Pathway

**Figure 5:**
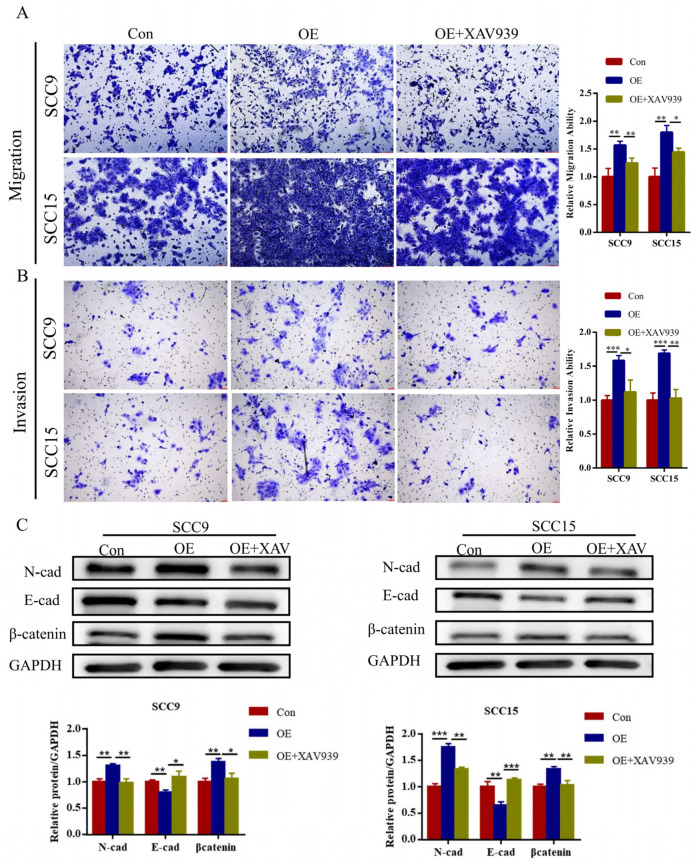
DEPDC1 promoted OSCC progression via the WNT/β-catenin signaling pathway. (**A**,**B**), Overexpression of DEPDC1 enhanced the migration and invasion of SCC9 and SCC15, while XAV939, an inhibitor of the WNT/β-catenin signaling pathway, could weaken the migration (SCC9 *p* = 0.0039; *p* = 0.0081, SCC15 *p* = 0.0024; *p* = 0.0134) and invasion ability (SCC9 *p* = 0.0005; *p* = 0.0138, SCC15 *p* = 0.0005; *p* = 0.0011) after overexpression of DEPDC1. (**C**), Meanwhile, upregulation of DEPDC1 induced higher N-cad (SCC9 *p* = 0.0024; *p* = 0.0042, SCC15 *p* = 0.0002; *p* = 0.0012) and β-catenin expression (SCC9 *p* = 0.0021; *p* = 0.0114, SCC15 *p* = 0.0014; *p* = 0.0075) and lower E-cad expression (SCC9 *p* = 0.0047; *p* = 0.0131, SCC15 *p* = 0.0064; *p* = 0.0004), while the protein level could also be reversed by XAV939. N-cad: N-cadherin, E-cad: E-cadherin, Con: control, OE: overexpression of DEPDC1. Significant differences were considered at *p* < 0.05 *; *p* < 0.01 **; *p* < 0.001 ***.

GSEA showed that high DEPDC1 expression was related to activation of the WNT/β-catenin pathway (Figure 3D,E). To further determine the effect of DEPDC1 in OSCC, the overexpression vectors of DEPDC1 and their controls were transfected into OSCC cells (Figure S4C,D), followed by treatment with XAV939 (Selleck Chemicals, Chengdu, China), an inhibitor of the WNT/β-catenin pathway, at a concentration of 10 μM. The results indicated that overexpression of DEPDC1 promoted the migration and invasion of OSCC. However, the migration and invasion abilities were reversed by XAV939 (Figure 5A,B). Meanwhile, upregulation of DEPDC1 increased the protein levels of β-catenin and N-cadherin and decreased E-cadherin expression (Figure 5C). The protein levels were also reversed by XAV939.

### 2.6. DEPDC1 Is Related to Glucose Uptake and Accumulation of Lactate in OSCC

**Figure 6:**
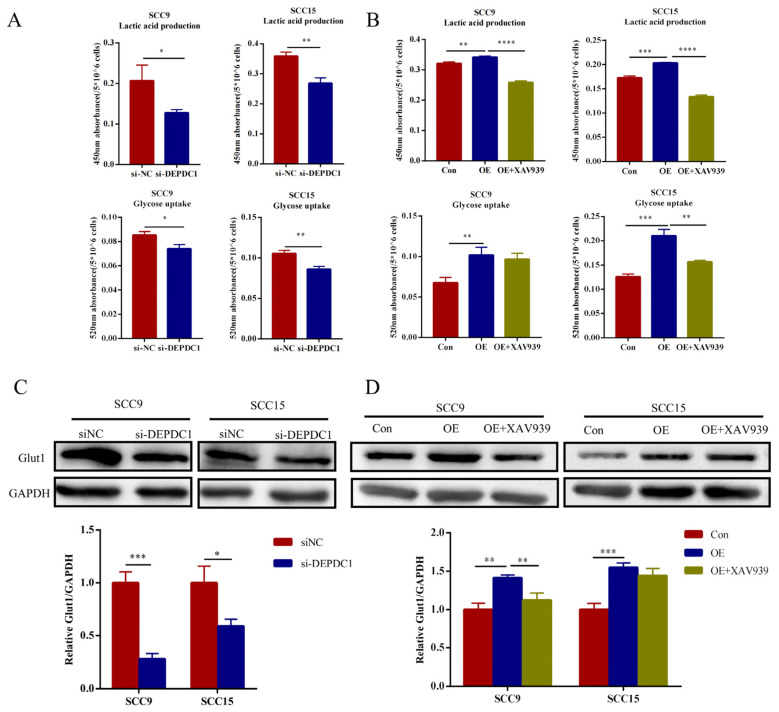
DEPDC1 promoted the aerobic glycolysis of OSCC. (**A**), Knockdown of DEPDC1 showed lower accumulation of lactate (SCC9 *p* = 0.0251, SCC15 *p* = 0.0023) and glucose uptake (SCC9 *p* = 0.0121, SCC15 *p* = 0.0035) in OSCC. (**B**), Lactic acid production (SCC9 *p* = 0.0051; *p* < 0.0001, SCC15 *p* = 0.0002; *p* < 0.0001) and glucose uptake (SCC9 *p* = 0.0067; *p* = 0.4904, SCC15 *p* = 0.0005; *p* = 0.0023) induced by DEPDC1 could be inhibited by XAV939. (**C**,**D**), Glut1 protein levels after knockdown (SCC9 *p* = 0.0004, SCC15 *p* = 0.0140) or overexpression (SCC9 *p* = 0.0013; *p* = 0.0072, SCC15 *p* = 0.0007; *p* = 0.1741) of DEPDC1 in SCC9 and SCC15. Con: control, OE: overexpression of DEPDC1. Significant differences were considered at *p* < 0.05 *; *p* < 0.01 **; *p* < 0.001 ***; and *p* < 0.0001 ****.

To explore whether DEPDC1 influences aerobic glycolysis, we detected the glucose uptake and lactate production in OSCC cells after knockdown or overexpression of DEPDC1. As shown in the figure, knockdown of DEPDC1 decreased the glucose uptake and accumulation of lactate (Figure 6A). This phenomenon was reversed after overexpression of DEPDC1. Nevertheless, the activation of aerobic glycolysis by upregulated DEPDC1 was abolished following treatment with XAV939 (Figure 6B). In addition, knockdown of DEPDC1 decreased the expression of glucose transporter 1 (Glut1), a major glucose transporter, while overexpression of DEPDC1 enhanced the Glut1 protein level, which was also abolished by XAV939 (Figure 6C,D).

## 3. Discussion

Generally, cells depend on mitochondrial oxidative phosphorylation to produce energy for cellular processes. However, cancer cells always demand large amounts of energy for their fast proliferation and metastasis [15]. Therefore, cancer cells reprogram metabolic pathways including aerobic glycolysis, glutamine metabolism, and fatty acid metabolism [4]. Among them, aerobic glycolysis, also called the “Warburg effect”, is a common method of metabolic reprogramming [7]. This process leads to an increase in glucose uptake, accumulation of lactate and faster production of ATP, which plays a crucial role in tumorigenesis and tumor progression [6]. In addition, glycolysis helps cancer cells survive under cellular stress and accelerated aerobic glycolysis is related to the treatment resistance of cancers [16]. Hence, these mechanisms need to be further explored in OSCC, which might be helpful for improving prognosis.

Owing to the lack of effective early biomarkers, the 5-year overall survival of OSCC is still poor. Recently, a number of studies have indicated that aerobic glycolysis is associated with the proliferation [17], invasion [18], metastasis [19] and EMT [20] of OSCC. Therefore, it may be appropriate to explore glycolysis-related pathways to screen for potential biomarkers of OSCC. In our study, two active glycolysis-related pathways were filtered following extraction of 255 core genes and 66 DEGs. The hallmark-glycolysis gene set includes genes encoding proteins involved in glycolysis and gluconeogenesis, while reactome-glycolysis is related to glycolysis and canonical pathways. According to the DEGs from two gene sets, a Cox risk model including DDIT4, DEPDC1, and SLC16A3 was constructed, which predicted the prognosis of OSCC patients accurately. In addition, the risk score might be an independent prognostic factor, indicating that the three genes might play a crucial carcinogenic role in the progression of OSCC. DDIT4, located in the cytosol, is related to the response to hypoxia, which plays a vital role in aerobic glycolysis in the tumor microenvironment. Recently, Han et al. also showed that DDIT4 could predict the prognosis of OSCC patients [21]. In addition, DDIT4 is known to participate in a variety of cancers. In colorectal cancer, DDIT4 has been identified as an advanced stage and metastasis biomarker [22]. Overexpression of DDIT4 could accelerate the proliferation and tumorigenesis of gastric cancer. Generally, inhibition of DDIT4 inactivated P53 signaling pathways and mitogen-activated protein kinase (MAPK) activity, which was associated with drug resistance to 5-fluorouracil [23]. In addition, DDIT4 expression could be induced by nicotinamide nucleotide transhydrogenase antisense RNA 1 (NNT-AS1)/miR-496 axis in prostate cancer and played a carcinogenic role in proliferation and migration [24]. Similarly, SLC16A3, also known as monocarboxylate transporter 3 (MCT3) or MCT4, was associated with lactate and pyruvate transport across plasma membranes, which played a crucial role in diverse malignant tumors. For instance, SLC16A3, mediated by LINC00035/CCAAT enhancer binding protein beta (CEBPB), enhanced the glycolysis and promoted the development of ovarian cancer [25]. Levels of aberrant N6−methylation of adenosine (m6A) at SLC16A3 were also relevant to the efficacy of immunotherapy in melanoma [26]. In the present study, DEPDC1 was identified as a glycolysis-related biomarker via integrated bioinformatics. DEPDC1 is a newly identified cancer-associated gene that is part of a transcription repressor complex and related to cellular processes such as the cell cycle, transcription, mitosis and apoptosis [27]. Recently, DEPDC1 was identified as participating in tumorigenesis and cancer progression. Guo et al. indicated that DEPDC1 was overexpressed in OSCC tissues and related to overall survival [28]. They also indicated that DEPDC1 promoted the proliferation of OSCC via inhibiting cytochrome P450 family 27 subfamily B member 1 (CYP27B1) expression [28]. In addition, Qiu et al. showed that DEPDC1 could facilitate development and metastasis of OSCC [29]. In our study, we demonstrated that DEPDC1 might target the WNT/β−catenin signaling pathway, and then promoted the migration, invasion, and aerobic glycolysis of OSCC. Geng et al. also demonstrated that DEPDC1 could promote nephroblastoma progression through the WNT/β-catenin signaling pathway [30]. Moreover, high DEPDC1 expression was also shown in advanced stage cancer and lymph nodes metastasis, suggesting that DEPDC1 might be an advanced biomarker in OSCC. Unfortunately, we failed to validate these data in real patient samples. Generally, there may be a small difference between the database and real patients. Therefore, a further study should be done to clarify their role in OSCC. In addition, DEPDC1 is known to be associated with various biological processes of other cancers. For instance, DEPDC1 promoted cancer metastasis and differentiation via accelerating the cell cycle from G1 to S phase in gastric cancer [31]. In hepatocellular carcinoma, DEPDC1, as a metabolic gene related to glycolysis, has been validated as a contributor to the reconstruction of the tumor microenvironment [32]. In addition, high DEPDC1 expression mediated by LincRNA regulator of reprogramming (Linc-ROR) accelerated the development of hepatocellular carcinoma and angiogenesis [33]. Meanwhile, DEPDC1 also promoted chemotherapy resistance [34]. These results suggest that DEPDC1 might be a potential therapeutic target.

In our study, GSEA showed that DEPDC1 was relevant to various signaling pathways in OSCC. Recently, Zhao et al. demonstrated that DEPDC1 overexpression led to a significant promotion of proliferation by regulating the cell cycle in breast cancer, and that DEPDC1 was associated with a high activation of the PI3K-AKT-mTOR signaling pathway [35]. In addition, DEPDC1 could interact with E2F1 and promote its transcriptional activity in prostate cancer, resulting in the activation of the E2F signaling pathway to regulate the G1/S phase cell cycle transition and then increase cell proliferation [36]. In nasopharyngeal carcinoma, DEPDC1 knockdown downregulated various downstream targets, such as c−Myc, BCL2, MMP2 and MMP9, which participated in proliferation, tumorigenesis, and metastasis [37], suggesting that DEPDC1 might be a vital factor in these biological processes. In OSCC, higher activation of the PI3K-AKT-mTOR pathway was reported to accelerate progression by increasing aerobic glycolysis [38]. Similarly, the E2F signaling pathway was also highly active in OSCC tissues, and this high activation drove radioresistance in OSCC patients, resulting in worse recurrence-free survival of radiotherapy patients [39]. Moreover, many studies suggest that adipogenesis [40], fatty acid metabolism [41], the MYC pathway [42], and the MTORC1 pathway [43] play a crucial role in the initiation and progression of OSCC. In conclusion, all of these pathways were remarkably relevant to OSCC initiation, progression, and therapy resistance. DEPDC1 was identified as activating these pathways through GSEA. Therefore, DEPDC1 is expected to be a novel biomarker and therapeutic target in OSCC. 

## 4. Materials and Methods

### 4.1. Data Acquisition from TCGA

TCGA is a landmark cancer genomics program which contains gene expression profiles and clinical data for 33 cancers [44]. All of the OSCC data were downloaded from the TCGA database. A total of 351 samples including 319 OSCC samples and 32 normal controls were obtained. Two OSCC samples were excluded because of insufficient clinical data.

### 4.2. Gene Set Enrichment Analysis (GSEA)

A total of four glycolysis-related pathways (biocarta-glycolysis-pathway, kegg-glycolysis-gluconeogenesis, hallmark-glycolysis, reactome-glycolysis) were obtained from the GSEA website (http://www.gseamsigdb.org/gsea/, accessed on 30 September 2021). Subsequently, GSEA was used to screen the activated glycolysis-related gene sets in OSCC. Furthermore, the glycolysis-related genes were extracted from the activated glycolysis-related gene sets and then we carried out differentially expressed analysis. In addition, GSEA was also used to investigate the potential pathways which were activated by DEPDC1 in OSCC.

### 4.3. Identification of Potential Prognostic Biomarker

Based on the differentially expressed genes (DEGs) from the glycolysis−related gene sets, univariate and multivariate Cox regression analyses were used to construct the Cox risk model and screen potential biomarkers to predict the prognosis of OSCC patients. Furthermore, the risk score of each patient was calculated on the basis of gene expression profiles, and independent prognosis analyses were carried out based on clinical parameters and risk score.

### 4.4. Survival Analysis

According to the risk score of each patient, OSCC samples were divided into high and low risk groups. Combined with the survival data in TCGA, survival analyses with the log rank *p* test were applied to verify the accuracy of the Cox model in R software with the survival package (Version R-4.2.1, Chengdu, China).

### 4.5. Cell Culture

The human OSCC cell lines SCC9 and SCC15 were purchased from ATCC. Cells were cultured in DMEM (Gibco, Cat#11995500TB, Chengdu, China) with 10% fetal bovine serum (ExCell Bio, Inc., Chengdu, China) and incubated at 37 °C with 5% CO_2_. The medium of all cells was exchanged about every 1–2 days according to the cell density. 

### 4.6. RNA Isolation and qRT-PCR

The protocol for RNA isolation and quantitative real-time PCR (qRT-PCR) has been described previously [45]. Briefly, total RNA was extracted from OSCC cells with TRIZOL reagent (Vazyme, Chengdu, China) and then reversed to cDNA. Subsequently, relative expression profiles of target genes were detected by qRT-PCR with ChamQ Universal SYBR qPCR Master Mix kit (Vazyme Biotech Co., Ltd., Chengdu, China). The primer sequences were as follows: DEPDC1 forward, 5′-ATGCGTATGATTTCCCGAATGAG-3′; DEPDC1 reverse, 5′-CACAGCATAACACACATCGAGAA-3′; GAPDH forward, 5′-CGCTGAGTACGTCGTGGAGTC-3′; and GAPDH reverse, 5′-GCTGATGATCTTGAGGCTGTTGTC-3′.

### 4.7. Cell Transfection

The small interfering RNAs (siRNAs) and DEPDC1 expression vectors were designed and synthesized in TINGKE (Cheng, China). According to the lipofectamine 3000 protocol (Invitrogen, Cat# L3000-015, Chengdu, China), cells were seeded in 6-well or 12-well plates for 12–24 h at a density of nearly 70–90%, and approximately 2500 ng siRNAs or DEPDC1 overexpression vectors were transfected into OSCC cells. The transfection system would last for 8–12 h, after which the medium was exchanged with fresh medium. After incubation for 2–4 days, RNA and protein were collected for analysis. The siRNA sequence of DEPDC1 was as follows: sense 5′-3′: CGCAGCCCUCUUGCUAUUTT, antisense 5′-3′: AAUAGCAUAGAGGGCUGCGTT. 

### 4.8. Transwell Assay

The protocol for the transwell assay has been described previously [46]. Briefly, 600 μL DMEM with 10% FBS was placed into the bottom wells, and about 5 × 10^4^ cells resuspended in 200 μL serum-free medium were put in the upper chambers. In addition, the invasion assay was performed with 100 μL 1:8 diluted matrigel in the upper chamber. The device was cultured at 37 °C with 5% CO_2_ for 24–72 h. Finally, the cells in the upper chamber were removed and the migration and invasion cells were stained with crystal violet.

### 4.9. Glucose Uptake and Lactate Detection

The cells of glucose uptake and lactate were detected using a detection kit (Cat#ADS-W-TDX002, ADS-W-T009-96, Chengdu, China) following the manufacturer’s instructions. Briefly, the same amounts of cells, about 5 × 10^6^ cells, were collected to obtain intracellular glucose and lactate. Cells were broken up by cell ultrasound equipment at 200 W for 3 s, with a 10 s pause between each cycle, for a total of 30 times, and then centrifuged at 12,000 rpm and 4 °C for 10 min to collect the supernatant. the absorbance of the supernatant at 450 nm (lactic acid production) and 520 nm (glucose uptake) were determined by a microplate reader. Higher absorbance meant a higher level of glucose uptake and accumulation of lactate.

### 4.10. Western Blotting

The Western blot methods have been elaborated upon [46]. In short, the proteins of the OSCC cell lines were extracted, separated by polyacrylamide gel electrophoresis (PAGE), and transferred to PVDF membranes which were then sealed in 5% skim milk for 1 h at room temperature. Subsequently, the membranes were incubated overnight with primary antibodies at 4 °C and second antibodies for 1 h at room temperature. Finally, ECL was used to analyze the protein level. The antibody information was as follows: GAPDH (Ray ANTIBODU, 1:2000, Cat#RM2002, Chengdu, China); DEPDC1 (Bioss, 1:1000, Cat#bs-6525R, Chengdu, China); N-cadherin (Proteintech, 1:2000, Cat# 22018-1-AP, Chengdu, China); E-cadherin (Proteintech, 1:2000, Cat#20874-1-AP, Chengdu, China); Glut1 (Wanleibio, 1:1000, Cat#WL03141, Chengdu, China); and β-catenin (Wanleibio, 1:1000, Cat#WL0962a, Chengdu, China).

### 4.11. Statiscal Analysis

SPSS software (IBM, Version 23.0, Armonk, NY, USA) was used to perform statistical analysis. The difference between two groups was investigated with a Student’s *t*-test, and the difference in one factor among multiple groups was explored with a one-way ANOVA. Results were expressed as the mean ± SD (standard deviation). Significant differences were considered at *p* < 0.05 *; *p* < 0.01 **; *p* < 0.001 ***; and *p* < 0.0001 ****.

## 5. Conclusions

A total of two glycolysis-related gene sets with aberrant activity in OSCC were identified following extraction of the core genes. Subsequently, we tried to construct a Cox model which could predict the prognosis of OSCC patients more accurately, and found that the risk score according DEPDC1, DDIT4 and SLC16A3 expression profiles might be an independent prognostic factor, indicating that these three genes might play a crucial role in the prognosis of OSCC. Moreover, DEPDC1 was identified as accelerating aerobic glycolysis, migration, and invasion via the WNT/β-catenin signaling pathway in vitro. Meanwhile, DEPDC1 was reported to be relevant to various cancer associated pathways. Our findings, along with previous reports, suggest that DEPDC1 might be a promising novel biomarker and therapeutic target in OSCC.

## Figures and Tables

**Table 1:** The core genes screened out from 2 glycolysis-related pathways.

AAAS	B4GALT2	CTH	GALE	HAX1	MDH2	NUP210	PFKP	PPP2R5D	SLC37A4
ABCB6	B4GALT4	CXCR4	GALK1	HDLBP	ME1	NUP214	PGAM1	PRKACA	SOD1
ADORA2B	B4GALT7	CYB5A	GALK2	HK1	ME2	NUP35	PGAM2	PRKACB	SOX9
ADPGK	BIK	DCN	GAPDH	HK2	MED24	NUP37	PGK1	PRKACG	SPAG4
AGL	BPGM	DDIT4	GAPDHS	HK3	MERTK	NUP43	PGK2	PRPS1	SRD5A3
AGRN	BPNT1	DEPDC1	GCK	HMMR	MET	NUP50	PGLS	PSMC4	STC1
AK3	CACNA1H	DLD	GCKR	HOMER1	MIF	NUP54	PGM2	PYGB	STC2
AK4	CAPN5	DPYSL4	GCLC	HS2ST1	MIOX	NUP58	PGM2L1	PYGL	STMN1
AKR1A1	CASP6	DSC2	GFPT1	HS6ST2	MPI	NUP62	PGP	QSOX1	TALDO1
ALDH7A1	CD44	ECD	GLCE	HSPA5	MXI1	NUP85	PHKA2	RAE1	TFF3
ALDH9A1	CDK1	EFNA3	GLRX	IDH1	NANP	NUP88	PKLR	RANBP2	TGFA
ALDOA	CENPA	EGFR	GMPPA	IDUA	NASP	NUP93	PKM	RBCK1	TGFBI
ALDOB	CHPF	EGLN3	GMPPB	IER3	NDC1	NUP98	PKP2	RPE	TKTL1
ALDOC	CHPF2	ELF3	GNE	IGFBP3	NDST3	P4HA1	PLOD1	RRAGD	TPBG
ALG1	CHST1	ENO1	GNPDA1	IL13RA1	NDUFV3	P4HA2	PLOD2	SAP30	TPI1
ANG	CHST12	ENO2	GNPDA2	IRS2	NOL3	PAM	PMM2	SDC1	TPR
ANGPTL4	CHST2	ENO3	GOT1	ISG20	NSDHL	PAXIP1	POLR3K	SDC2	TPST1
ANKZF1	CHST4	ERO1A	GOT2	KDELR3	NT5E	PC	POM121	SDC3	TSTA3
ARPP19	CHST6	EXT1	GPC1	KIF20A	NUP107	PDK3	POM121C	SDHC	TXN
ARTN	CITED2	EXT2	GPC3	KIF2A	NUP133	PFKFB1	PPFIA4	SEC13	UGP2
AURKA	CLDN3	FAM162A	GPC4	LCT	NUP153	PFKFB2	PPIA	SEH1L	VCAN
B3GALT6	CLDN9	FBP2	GPI	LDHA	NUP155	PFKFB3	PPP2CA	SLC16A3	VEGFA
B3GAT1	CLN6	FKBP4	GPR87	LDHC	NUP160	PFKFB4	PPP2CB	SLC25A10	VLDLR
B3GAT3	COG2	FUT8	GUSB	LHPP	NUP188	PFKL	PPP2R1A	SLC25A13	XYLT2
B3GNT3	COL5A1	G6PD	GYS1	LHX9	NUP205	PFKM	PPP2R1B	SLC35A3	ZNF292
B4GALT1	COPB2	GAL3ST1	GYS2	MDH1					

**Table 2:** The downregulated glycolysis-related genes in OSCC.

Gene	logFC	*p*-Value	Gene	logFC	*p*-Value
FBP2	−5.0625	<0.0001	CAPN5	−1.8001	<0.0001
ENO3	−4.3165	0.0003	AGL	−1.4033	0.0002
GYS2	−3.2913	<0.0001	GCKR	−1.361	0.0003
CHST4	−2.7761	0.0031	GCKR	−1.361	0.0003
PFKFB1	−2.5352	0.0002	ELF3	−1.2759	<0.0001
PFKFB1	−2.5352	0.0002	GOT1	−1.1533	<0.0001
GAPDHS	−2.4599	0.0003	GNE	−1.1137	0.0011
RRAGD	−2.414	<0.0001	ALDH9A1	−1.0858	<0.0001
TFF3	−2.3824	<0.0001	ANG	−1.0821	0.001
CLDN3	−2.0254	0.0124			

**Table 3:** The upregulated glycolysis-related genes in OSCC.

Gene	logFC	*p* Value	Gene	logFC	*p* Value
NUP155	1.0005	<0.0001	CDK1	1.6558	<0.0001
XYLT2	1.022	<0.0001	EXT1	1.6601	<0.0001
BPGM	1.0342	<0.0001	P4HA2	1.69	<0.0001
SPAG4	1.045	<0.0001	DEPDC1	1.7072	<0.0001
GPC1	1.048	<0.0001	P4HA1	1.7279	<0.0001
TGFA	1.0601	<0.0001	TPBG	1.7836	<0.0001
NUP107	1.0995	<0.0001	HK3	1.8105	<0.0001
PFKFB4	1.1089	<0.0001	VCAN	1.8404	<0.0001
KDELR3	1.1278	<0.0001	AGRN	1.8455	<0.0001
ISG20	1.1342	0.0001	KIF20A	1.8507	<0.0001
EXT2	1.1395	<0.0001	PLOD2	1.8644	<0.0001
DDIT4	1.162	<0.0001	IGFBP3	1.9107	<0.0001
B4GALT4	1.1713	<0.0001	HMMR	1.9118	<0.0001
VEGFA	1.1938	<0.0001	NT5E	2.1584	<0.0001
CHST1	1.2133	0.0001	AURKA	2.2105	<0.0001
SLC16A3	1.2878	<0.0001	CENPA	2.2121	<0.0001
CHPF	1.2915	<0.0001	CHST2	2.4394	<0.0001
STC1	1.3686	0.0001	ENO2	2.6407	<0.0001
MET	1.3857	<0.0001	STC2	2.9567	<0.0001
STMN1	1.4038	<0.0001	COL5A1	3.5266	<0.0001
EGLN3	1.4516	<0.0001	ARTN	3.6158	<0.0001
PYGL	1.4539	<0.0001	TGFBI	4.0522	<0.0001
EGFR	1.4653	<0.0001	MIOX	4.2703	<0.0001
TPST1	1.5324	<0.0001	LHX9	4.6398	<0.0001
PLOD1	1.5884	<0.0001			

## Data Availability

All the data presented in this study are available on request from the corresponding author. Publicly available data were obtained from official TCGA (https://portal.gdc.cancer.gov/, accessed on 30 September 2021) and GSEA websites (https://www.gsea-msigdb.org/gsea/index.jsp, accessed on 30 September 2021).

## Supplementary Materials

The following supporting information can be downloaded at: https://www.mdpi.com/article/10.3390/ijms24031992/s1.
