# Supplementary material for: Glycolysis-Related Gene Analyses Indicate That DEPDC1 Promotes the Malignant Progression of Oral Squamous Cell Carcinoma via the WNT/β-Catenin Signaling Pathway
Source: Int J Mol Sci. 2023 Jan 19;24(3):1992. doi: 10.3390/ijms24031992 (PMC9916831; doi:10.3390/ijms24031992)
Supplement: Supplementary file 1 [file ijms-24-01992-s001.zip › ijms-2133707-supplementary.pdf]

Figure S1

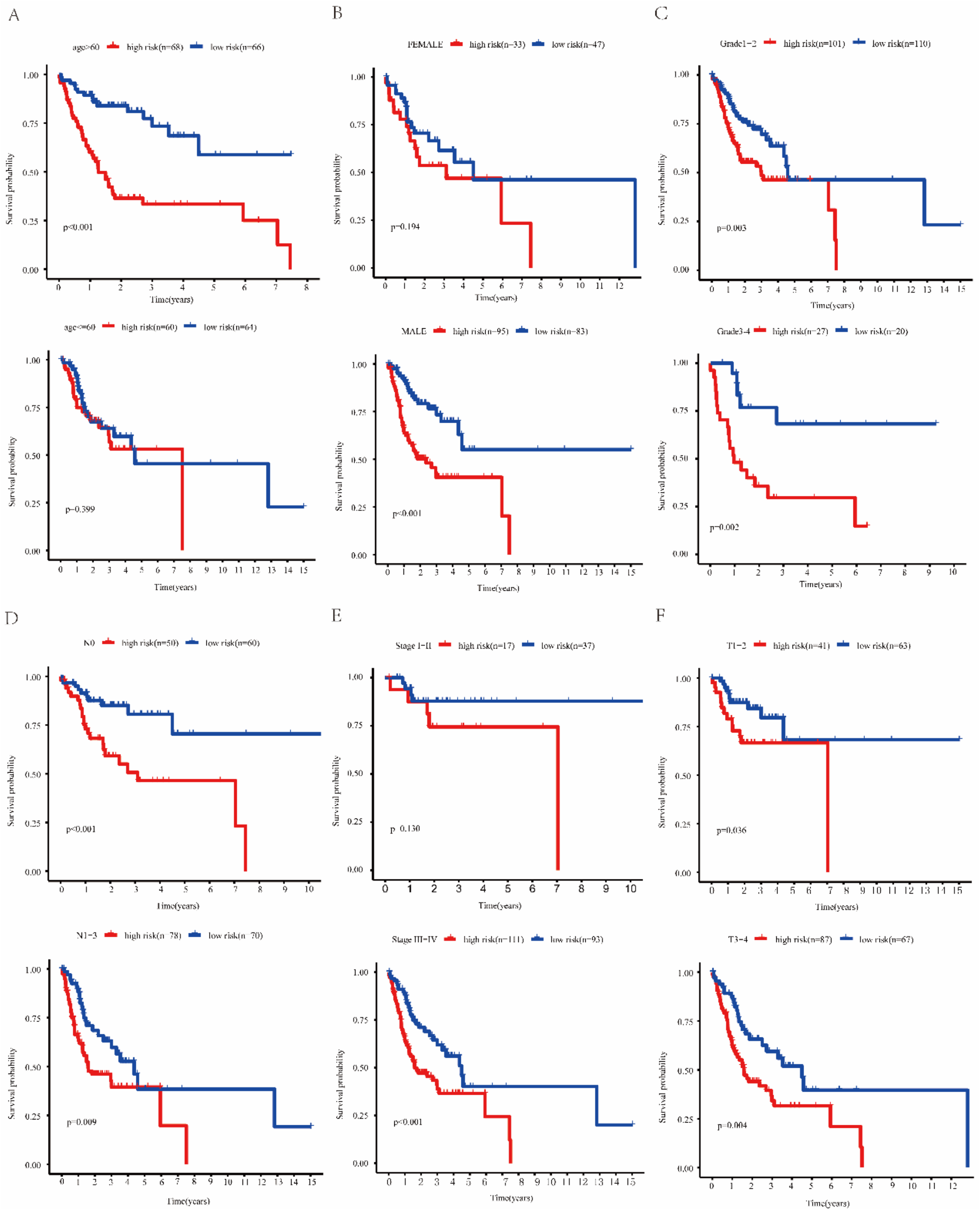

Figure S1 Stratification analyses of A; age, B; gender, C; grade, D; stage and E; T classification, F; N classification in TNM system combined with survival analysis were used to explore the prognostic value of risk cox formula.

Figure S2

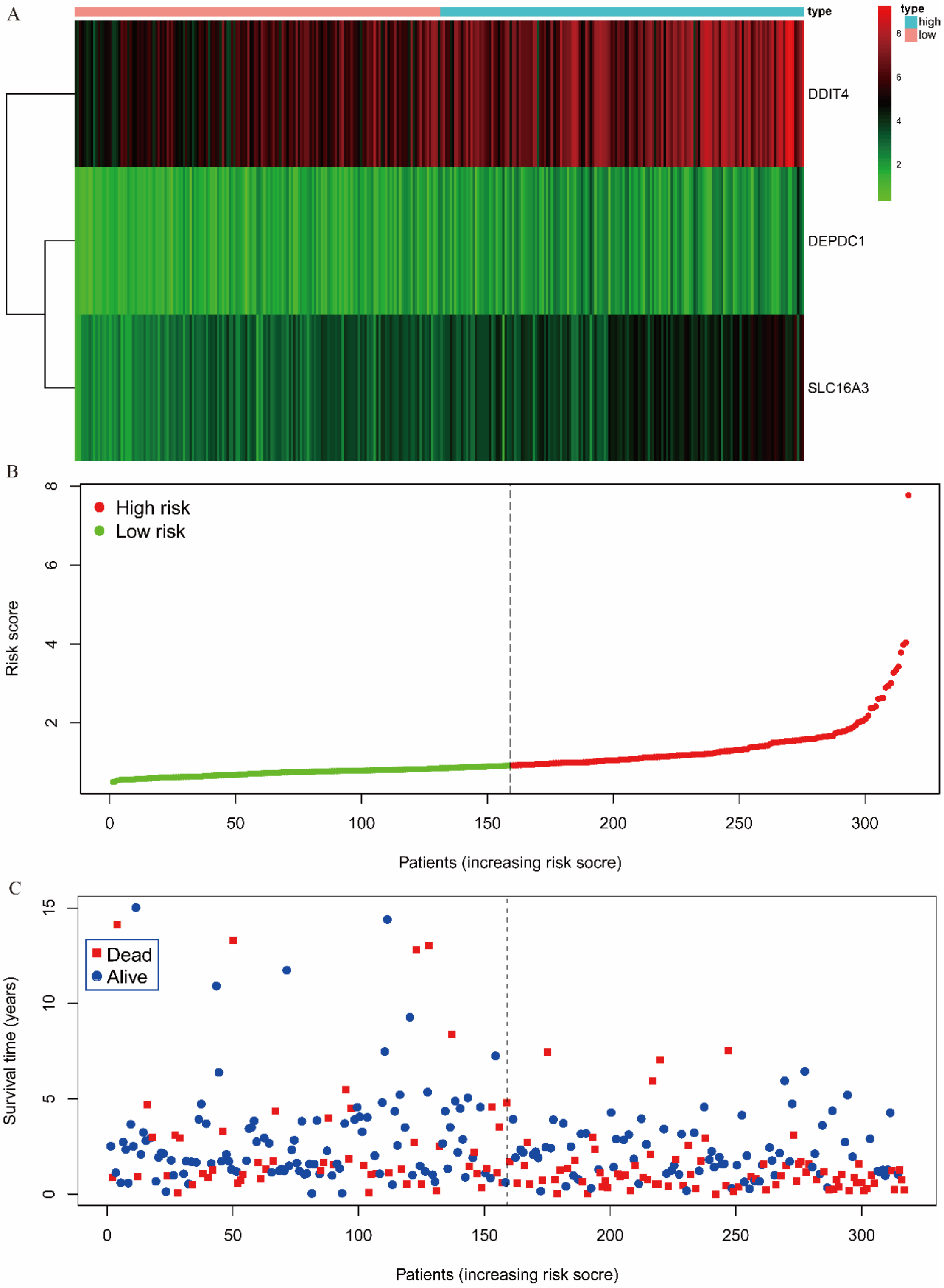

Figure S2 Visualization of prognostic model. A; Gene expression profiles in risk formula. Green and red stand for low and high gene expression, respectively. Pink and blue stand for low and high risk group respectively. B, C; Combination of survival time and risk score. Horizontal axis and vertical axis stand for risk score and survival time respectively.

Figure S3

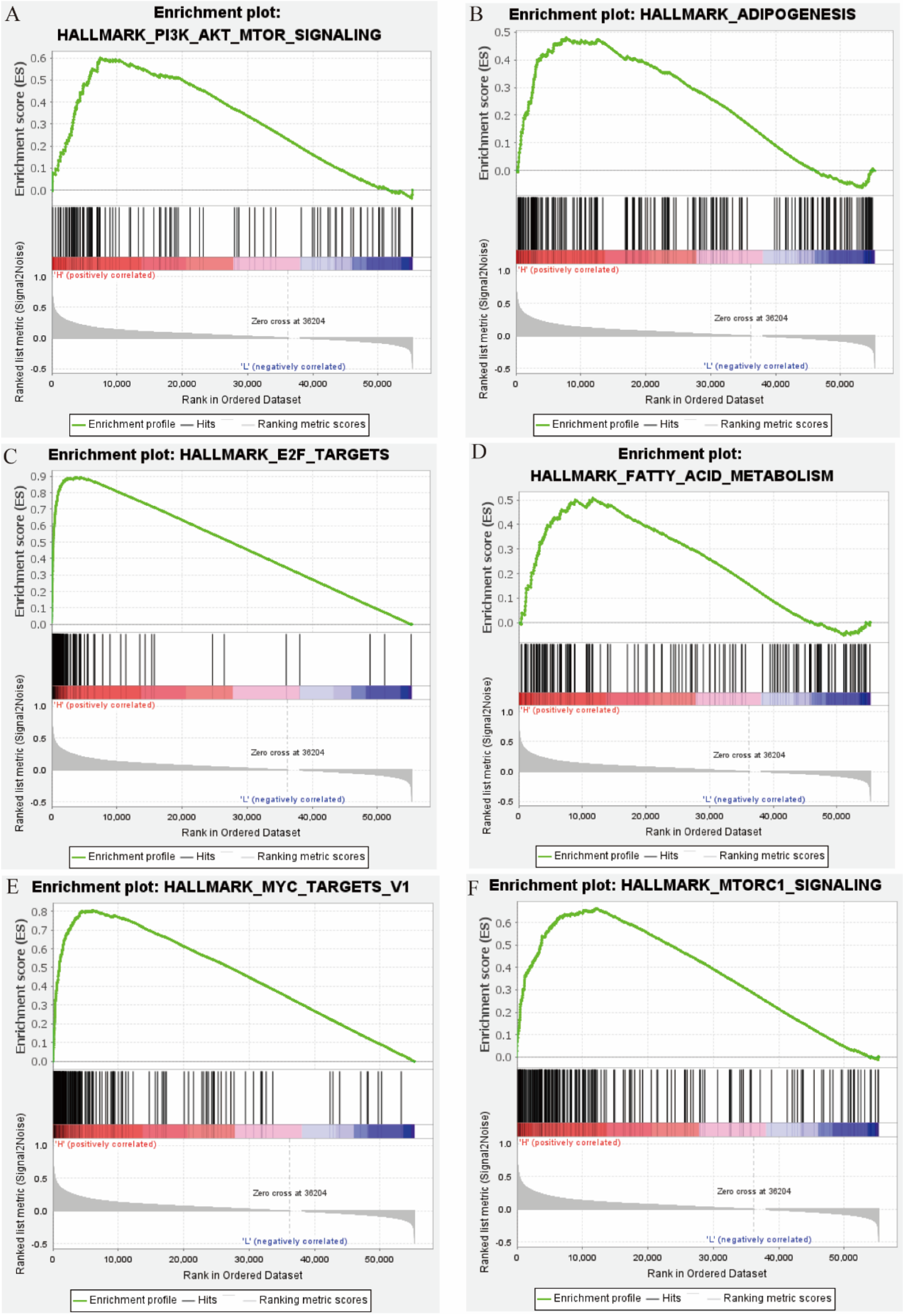

Figure S3 DEPDC1 was related to various pathways in OSCC. GSEA was used to further explore the role of DEPDC1 in OSCC. higher depdc1 was associated with higher activation PI3K-AKT-MTOR-signaling (A), adipgenesis (B), E2F-targets (C), fatty-acid-metabolism (D), MYC-targets (E) and MTORC1-signaling (F).

Figure S4

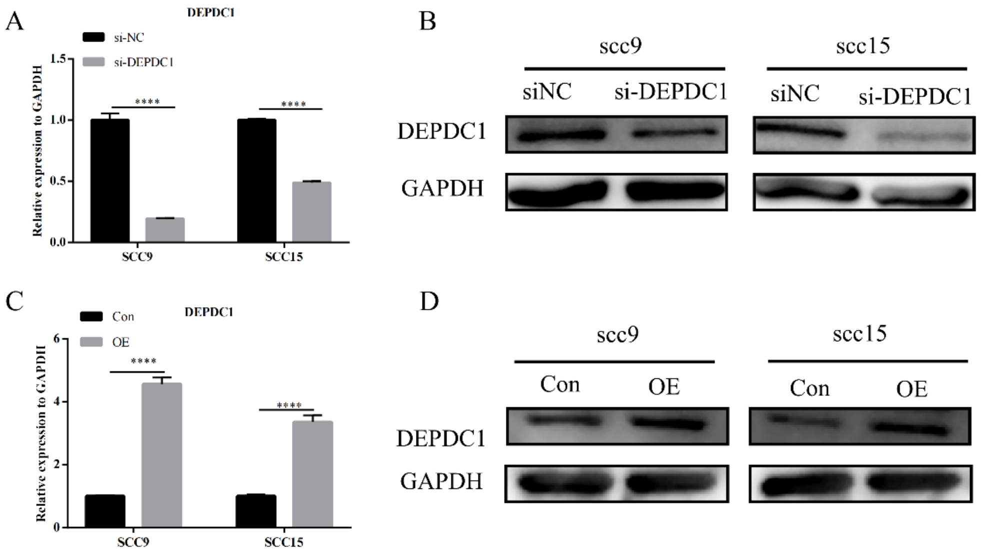

Figure S4 The efficiencies of knockdown (SCC9 p<0.0001, SCC15 p<0.0001) and overexpression (SCC9 p<0.0001, SCC15 p<0.0001) of DEPDC1 were detected by qRT-PCR (A;C) and Western blot (B;D).

Table S1

Univariate Cox regression analysis

Gene	HR	HR.95L	HR.95H	Cox p-value
HMMR	1.055617293	1.009592348	1.103740407	0.0173
STC2	1.030630074	1.00569649	1.056181821	0.0158
DDIT4	1.002705713	1.001223766	1.004189855	0.0003
DEPDC1	1.081286039	1.014198308	1.152811526	0.0168
SLC16A3	1.038753593	1.018766923	1.059132371	0.0001
AURKA	1.030609225	1.00648701	1.055309571	0.0126

Table S2

Risk formula according to Multivariate Cox regression analysis

Id	Coef	HR
DDIT4	0.002204596	1.002207028
DEPDC1	0.078764107	1.081949068
SLC16A3	0.031686448	1.032193808
